# Postoperative Chronic Hypoparathyroidism and Quality of Life After Total Thyroidectomy

**DOI:** 10.1002/jbm4.10479

**Published:** 2021-03-16

**Authors:** Camilla Uhre Jørgensen, Preben Homøe, Morten Dahl, Mette Friberg Hitz

**Affiliations:** ^1^ Department of Otorhinolaryngology and Maxillofacial Surgery Zealand University Hospital (ZUH) Køge Denmark; ^2^ Department of Medical Endocrinology Zealand University Hospital (ZUH) Køge Denmark; ^3^ Department of Clinical Medicine, Faculty of Health and Medical Sciences University of Copenhagen Copenhagen Denmark; ^4^ Department of Clinical Biochemistry Zealand University Hospital (ZUH) Køge Denmark

**Keywords:** CALCIUM, PARATHYROIDEA, QoL, RAND‐36, THYROIDECTOMY

## Abstract

Chronic hypoparathyroidism (HypoPT) is a common complication after total thyroidectomy and it impacts affected patients' quality of life (QoL). This study aimed to assess the QoL in patients with chronic HypoPT independently from their concurrent hypothyroidism and other comorbidities. For this purpose a follow‐up study was performed, including 14 patients who developed chronic HypoPT after total thyroidectomy and 28 age‐ and sex‐matched patients who had intact parathyroid function after total thyroidectomy. We used the RAND Short Form 36 Health Survey (SF‐36) to compare the QoL between patients with or without chronic HypoPT. Chronic HypoPT patients had lower QoL scores in all domains of the RAND‐SF‐36 questionnaire and significant impairment in six of eight domains after adjustment for relevant confounders. They were more often operated because of a toxic diagnosis (*p* = .01), often being Graves disease. Additionally adjusting for surgical indications resulted in three of eight domains being significant affected. Chronic HypoPT is associated with significantly impairment of QoL, independently of the concurrent disease of hypothyroidism, comorbidities, and prospective values of TSH and serum (se)‐ionized‐Ca^++^. There is a need for more focus and better treatment of patients experiencing chronic HypoPT after surgery. © 2021 The Authors. *JBMR Plus* published by Wiley Periodicals LLC on behalf of American Society for Bone and Mineral Research.

## Introduction

Postoperative hypoparathyroidism (HypoPT) is an endocrine disorder caused by iatrogenic injury to the parathyroid glands during thyroid surgery. HypoPT is characterized by an inappropriately low level of circulating parathyroid hormone (PTH), resulting in hypocalcemia.^(^ ^1^ ^)^

The reported incidence of postoperative chronic HypoPT 6 months after thyroidectomy is highly variable, ranging from 0% to 17%, depending on the methods used to define HypoPT in the specific studies.^(^ ^2^, ^3^, ^4^, ^5^, ^6^ ^)^

Chronic HypoPT is a complex condition to treat. Current treatment involves supplementation with calcium and active vitamin D analogues, which makes chronic HypoPT the only remaining endocrine disorder where standard treatment does not consist of replacing the missing hormone.^(^ ^1^, ^7^, ^8^ ^)^

In order to obtain normocalcemic levels in blood, patients with chronic HypoPT need lifelong medication. Despite this, patients often experience calcium fluctuations because of lack of direct and indirect actions of PTH, contributing to hypercalciuria and hyperphosphatemia. These changes are associated with severe long‐term complications,^(^ ^1^, ^7^, ^8^, ^9^ ^)^ particularly in the renal, neuropsychiatric, and musculoskeletal organ systems, resulting in impaired health‐related quality of life (QoL) in patients with chronic HypoPT.^(^ ^1^, ^9^, ^10^, ^11^ ^)^

It is noteworthy, that all patients with chronic HypoPT due to total thyroidectomy (TT) are also suffering from a concurrent hypothyroidism. This condition has previously been shown to independently affect the QoL,^(^ ^12^ ^)^ and its unclear whether QoL impairment in chronic HypoPT is due to insufficient production of PTH, thyroid hormones, or a combination. A few other studies^(^ ^11^, ^13^ ^)^ have sought to address this, but their results have been inconclusive.

The aim of this study is to test whether QoL is reduced in patients with chronic HypoPT independently from comorbid hypothyroidism. For this purpose we compared QoL scores in patients with postoperative chronic HypoPT after TT versus patients with intact parathyroid function after TT.

## Patients and Methods

### Study subjects

For this follow‐up study, we identified all patients who underwent TT due to a benign thyroid disorder at Zealand University Hospital (ZUH), Køge (Denmark) from 2013 to 2018.^(^ ^6^ ^)^ Data on year and type of surgery was drawn from the national Thykir database, which contains information regarding all thyroid procedures in Denmark since 2001, and supplemented by procedure codes (KBAA60 and KBAA60A). In our department, this also included completion thyroidectomy.

We invited all patients identified with postoperative chronic HypoPT at 6 months after TT, and of the 25 patients invited, 14 (56%) accepted the invitation to participate in this study. Those who declined to participate were more often males (54%), but did not differ in either geographical living area, year of surgery, or preoperative health status from the participants included in the study. For each patient with chronic HypoPT, we invited two age‐ and gender‐matched patients from the same study population, but without any postoperative disturbances in calcium homeostasis (without chronic HypoPT). Median duration since surgery was 41.5 months (range, 12–70).

In accordance with the European Society of Endocrinology Clinical Guideline,^(^ ^1^ ^)^ HypoPT was defined as hypocalcemia and inappropriately low serum (se)‐PTH due to insufficient production of PTH. Chronic HypoPT was confirmed if the condition persisted ≥6 months and/or if the patient needed continuous treatment with an active vitamin D to maintain normocalcemia. Hypocalcemia was defined as se‐ionized‐Ca^++^ <1.16 mmol/L in two consecutive measurements, in accordance with the lower limit of the local hospital laboratory.

Se‐ionized‐Ca^++^ was measured at latest the first day postoperatively after TT and then three times daily until stabilization or normal values were obtained. Patients in continuous need of treatment with an active vitamin D were followed up by endocrinologists. Attempts to reduce doses or stop treatment were standard procedure.

We further retrieved preoperative and postoperative se‐TSH, medical prescriptions for active vitamin D analogues, and data on the indication for surgery (toxic vs nontoxic diagnosis) from the patient health records. Surgical indications were most often pressure symptoms (including globus and respiratory complaints), and dysphagia due to either a toxic or nontoxic disease. Some patients being hyperthyroid preoperatively had surgery due to multiple relapse of toxicity and few were non‐tolerant to medicine.

### Inclusion/exclusion criteria

Patients with and without chronic HypoPT met the same inclusion and exclusion criteria. Inclusion criteria: patients (i) undergoing TT or completion thyroidectomy for a benign disorder performed at the Department of Otorhinolaryngology and Maxillofacial Surgery, ZUH Køge in the period June 1, 2013 to September 30; 2018, (ii) ≥18 years at the time of inclusion; and (iii) able to provide written informed consent to the study invitation. Exclusion criteria: patients with (i) a diagnosis of thyroid cancer; (ii) prior parathyroid disease; (iii) prior disturbances in calcium homeostasis, for example, hypocalcemia or estimated glomerular filtration rate (eGFR) <30 mL/min, or plasma creatinine >200 μmol/L; (vi) sarcoidosis; or (v) transient HypoPT disturbances lasting <6 months (transient HypoPT).

### Outcome measures and data collection

All included patients were invited to clinical evaluations at the department of Medical Endocrinology ZUH Køge, where prospective data collection took place. Two patients completed the data collection from home.

### Primary outcome

#### QoL

We used the RAND Short Form 36 Health Survey (SF‐36)^(^ ^14^ ^)^ to measure and compare QoL in patients with and without chronic HypoPT. The RAND SF‐36 consists of 36 questions and covers eight concepts of physical and mental health: physical functioning (PF), role physical (RP), bodily pain (BP), general health (GH), vitality (VT), social functioning (SF), role emotional (RE), and mental health (MH). By the RAND algorithm, the scores were recalculated and expressed into a 0 to 100 scale. Higher score indicates more favorable QoL. The validated Danish version of the RAND SF‐36 is a self‐reported questionnaire to be completed electronically.

### Secondary outcomes

#### Prospective biochemistry

Se‐ionized‐Ca^++^, PTH, 25‐Hydroxyvitamin D, phosphate, thyroid status (TSH, T4, T3), renal status (creatinine, eGFR), and liver status (alanine aminotransferase [ALAT], bilirubin, lactate dehydrogenase [LDH], basic phosphatase) were measured by standard hospital assays. All biochemical samples were taken after patient inclusion and measured from May 1, 2019 to September 1, 2019.

#### Medicine

All patients informed us of their daily medication consumption, which was verified in the individual patient health records.

#### Comorbidity

Age‐adjusted Charlson Comorbidity Index (ACCI) score was calculated to account for competing concurrent diseases, aside from the primary disease of interest. ACCI is a method of categorizing comorbidities based on a patient interview and the International Classification of Diseases (ICD). Each comorbidity was scored from 1 to 6 and for each decade >50 years, a score of 1 was added. In case of cancer or metastasis, points were only assigned if present ≤5 years.

### Ethical statement

We performed the study in accordance with the Declaration of Helsinki II. All participants provided written informed consent before any procedures took place. The study was approved by The Danish Data Protection Agency (REG‐015‐2019) and The Ethical Committee of Central Denmark (No. 66792).

### Statistics

Statistical analyses were conducted using R‐studio v. 1.2.1335 (R Foundation for Statistical Computing, Vienna, Austria; https://www.r-project.org/). The level of significance was defined as *p* < .05. Results are reported as *n* (%) or as median with range, as indicated. Comparison of groups was assessed by chi‐square test or Fisher's exact test for categorical variables, and two‐sample *t* test or Mann‐Whitney *U* test for continuous variables.

When assessing QoL score, we adjusted for age, ACCI, se‐TSH, se‐ionized‐Ca^++^, and/or indication for surgery using the analysis of covariance (ANCOVA). ACCI was transformed into a categorical variable consisting of integers (0 to ≥2).

## Results

### Characteristics

All 42 patients included were women (Table 1). Patients with chronic HypoPT had more often a toxic indication for surgery when compared to patients without chronic HypoPT (*p* = .01). Among patients with a toxic indication for surgery, 87.5% had Graves disease (data not shown).

**Table 1:** Clinical Characteristics of Patients With Chronic Hypoparathyroidism After Total Thyroidectomy

Characteristic	No chronic HypoPT (*n* = 28)	Chronic HypoPT (*n* = 14)	*p*
Females, *n* (%)	28 (100)	14 (100)	NA
Age (years), median (range)	53 (27–74)	44 (34–72)	NA
Indication for surgery, *n* (%)			**.01**
Toxic	7 (25)	9 (64.3)	
Nontoxic	21 (75)	5 (35.7)	
Medical treatment			
Levothyroxine, *n* (%)	27 (96.4)	14 (100)	1.00
Dose (μg/day), median (range)	129 (0–300)	168 (50–300)	.11
Alfacalcidol, *n* (%)	0	13 (93)	NA
Dose (μg/day), median (range)	0	1.5 (0–6)	NA
Calcium supplement, *n* (%)	14 (50)	14 (100)	**.001**
Dose (mg/day), median (range)	80 (0–800)	1400 (400–3700)	**<.001**
Vitamin D3 supplements, *n* (%)	18 (64.3)	13 (93)	.06
Dose (μg/day), median (range)	10 (0–76)	49 (0–114)	**.001**
ACCI score, *n* (%)			**.01**
0	9 (32)	10 (72)	
1	14 (50)	1 (7)	
≥2	5 (18)	3 (21)	

Values are *n* (%) or median (range). Bold values are significant at *p* < .05. Values of *p* by two‐sample *t* test, Mann‐Whitney *U* test, chi‐square test, or Fisher's excact as appropriate.Abbreviations: ACCI, age‐adjusted comorbidity score; HypoPT, hypoparathyroidism; NA, not applicable.

Patients with chronic HypoPT used more often daily calcium (100% vs 50%) and vitamin D3 supplements (93% vs. 64%) compared with patients without chronic HypoPT (Table 1). The median doses of daily calcium and vitamin D3 consumption were significantly higher among patients with chronic HypoPT. In addition, all of them, except one, received daily supplementation with an active vitamin D analogue in terms of Alfacalcidol (mean 1.7 μg/day) and all patients in both groups received standard levothyroxine for their hypothyroidism except one group who received desiccated thyroid extract.

ACCI was higher among patients without chronic HypoPT (*p* = .01).

Patients with chronic HypoPT had significantly lower serum PTH level, higher levels of serum 25‐hydroxyvitamin D and serum phosphate (Table 2). Some patients with chronic HypoPT were seen to have a minor residual parathyroid function (s‐PTH 1.6 pmol/L [range, 0.50–3.4 pmol/L]). These patients also presented with more irregular levels of se‐ionized‐Ca^++^, as indicated by the lower minimum value, as compared with patients without chronic HypoPT (range, 0.89–1.40 mmol/L vs. 1.17–1.35 mmol/L, lower limit of reference interval = 1.16 mmol/L).

**Table 2:** Biochemical Characteristics of Patients With Chronic Hypoparathyroidism After Total Thyroidectomy

Characteristic	No chronic HypoPT (*n* = 28)	Chronic HypoPT (*n* = 14)	*p*
Preoperative			
TSH (mIU/L)	0.77 (0.005–6.2)	0.58 (0.005–2.0)	.83
Prospective			
Calcium, ionized (mmol/L)	1.28 (1.17–1.35)	1.27 (0.89–1.40)	.18
PTH (pmol/L)	5.3 (2.3–9.6)	1.6 (0.50–3.4)	**<.001**
25‐Hydroxyvitamin D (nmol/L)	62 (29–125)	101 (34–146)	**<.01**
Phosphate (mmol/L)	1.06 (0.70–1.36)	1.13 (0.79–1.60)	**<.05**
Magnesium (mmol/L)	0.90 (0.79–0.99)	0.93 (0.73–1.00)	.68
eGFR (mL/min)	90 (65‐>90)	90 (55 to >90)	.70
TSH (mIU/L)	0.70 (0.005–19)	0.55 (0.005–17)	.94

Values are median (range). Bold values are significant at *p* < .05. Values of *p* by two‐sample *t* test or Mann‐Whitney *U* test. Prospective values were taken from May 1, 2019 to September 1, 2019.Abbreviations: eGFR, estimated glomerular filtration rate; HypoPT, hypoparathyroidism; PTH, parathyroid hormone; TSH, thyroid‐stimulating hormone.

Preoperative TSH was significantly lower (*p* < .01) in patients with a toxic surgical indication, compared with nontoxic (median 0.045 mIU/L [range, 0.005–2.00 mIU/L] vs. 0.87 mIU/L [range, 0.02–6.2 mIU/L]). Results not shown in table 2.

### QoL

**Figure 1:**
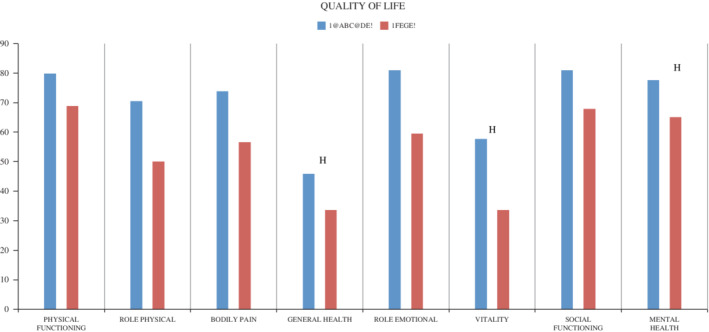
Unadjusted QoL scores by RAND SF‐36 questionnaire in patients with chronic hypoparathyroidism. QoL, quality of life; SF‐36, Short Form 36 Health Survey.

The scores for the eight QoL RAND‐SF‐36 measures were higher in patients with chronic HypoPT compared with patients without chronic HypoPT although not all comparisons reached statistical significance (Figure 1). After adjustment for age, comorbidities (ACCI), TSH and se‐ionized‐Ca^++^ patients with chronic HypoPT had significantly lower scores in the QoL domains: physical functioning (PF), role physical (RP), bodily pain (BP), general health (GH), vitality (VT), and mental health (MH) (Table 3). After additionally adjusting for surgical indications, chronic HypoPT patients were significantly affected in three of eight domains: role physical (RP), general health (GH), and vitality (VT) (data not shown). Comparing chronic HypoPT patients with a minor residual parathyroid function (s‐PTH >0.5 pmol/L; *n* = 9) to chronic HypoPT with no residual function (*n* = 4), 3 of 8 QoL domains were significantly affected in both groups, when comparing with patients without chronic HypoPT (Supplementary Table S1.)

**Table 3:** Adjusted QoL Scores by RAND‐SF‐36 Questionnaire in Patients With Chronic HypoPT

SF‐36 variables	No chronic HypoPT (*n* = 28)	Chronic HypoPT (*n* = 14)	Adjusted model 1^a^
Physical functioning	81.9 ± 3.7	66.7 ± 5.5	0.03
Role physical	80.8 ± 6.0	48.2 ± 12.1	0.02
Bodily pain	77.3 ± 5.0	54.9 ± 9.0	0.04
General health	46.9 ± 2.4	33.2 ± 3.3	<0.01
Role emotional	85.8 ± 5.9	61.9 ± 12.4	0.11
Vitality	58.2 ± 3.6	31.7 ± 7.4	<0.01
Social functioning	85.8 ± 3.6	70.9 ± 7.2	0.08
Mental health	77.3 ± 2.8	63.1 ± 4.4	0.02

Values are mean ± SD. Differences in QoL score were assessed using ANCOVA.Abbreviations: ACCI, age‐adjusted Charlson Comorbidity Index; ANCOVA, analysis of covariance; HypoPT, hypoparathyroidism; QoL, quality of life; SD, standard deviation; SF‐36, Short Form 36 Health Survey; TSH, thyroid‐stimulating hormone.

^a^ Adjusted for age, ACCI, TSH, and ionized calcium.

## Discussion

Patients with postsurgical HypoPT have an inappropriately low level of circulating parathyroid hormone (PTH). The chronic condition results in disturbances in calcium‐phosphate homeostasis, where patients often experience severe long‐term complications, including lower QoL. This study is one of the few to highlight the impact of chronic HypoPT on QoL, taking the surgical indication and comorbidities into account, in patients receiving conventional treatment after TT compared to patients without evidence of any parathyroid disturbances after TT.

We find in this study that patients who suffer from chronic HypoPT had lower QoL scores in all domains of the RAND‐SF‐36 questionnaire. Although not all comparisons reached statistical significance, our data suggests significant lower scores of QoL in six of eight domains among patients suffering from chronic HypoPT, compared with matched and clinically relevant patients without chronic HypoPT. These reductions are considerably higher than the minimal clinically important difference of proposed for RAND‐SF‐36 scores in clinical trials.^(^ ^15^ ^)^ After adjustment for patients' surgical indication (toxic/nontoxic diagnosis) significantly lower scores were observed only for the RP, GH, and VT domains; however, the trend remained the same, that all QoL domains were more reduced in patients with chronic HypoPT compared to patients without chronic HypoPT. Additionally, we compared chronic HypoPT patients with a minor residual parathyroid function (s‐PTH >0.5 pmol/L), to chronic HypoPT patients with no residual function, showing impairment of QoL in three of eight domains in both groups. Considering the small sample size (*n* = 9 and *n* = 5), we could not conclude any difference in QoL pattern regarding a residual parathyroid function.

Other studies using a SF‐36 questionnaire in the investigation of QoL present impairment in all eight domains,^(^ ^7^, ^16^ ^)^ whereas others reflect our result, only showing significant impairment in some domains, including PF, RP, BP, GH, VT, and SF,^(^ ^11^, ^17^, ^18^, ^19^ ^)^ roughly in accordance with our results. The studies reporting impairment in all eight domains compared their HypoPT patients to a general population and not to well‐matched controls. Other questionnaires (SCL‐90‐R and GBB‐24), as used by Arlt and colleagues,^(^ ^13^ ^)^ confirm the same pattern.

Compared to other chronic diseases, a Norwegian study^(^ ^16^ ^)^ reported QoL in HypoPT patients to be significantly lower than in patients with Addison's disease, and a systematic review^(^ ^17^ ^)^ reported an effect to the same extent as patients suffering from diabetes or heart diseases.

This study has not investigated the QoL score in the healthy Danish population. However, another Danish study^(^ ^11^ ^)^ also using the SF‐36 questionnaire reported significantly lower scores in seven of eight domains, when comparing HypoPT patients with healthy controls.

### Preoperative toxicity

Patients with and without chronic HypoPT were generally well matched and all had previously undergone TT, but chronic HypoPT patients were more often operated because of a toxic diagnosis. Patients with a toxic indication for surgery suffered mainly from Graves disease (87.5%). Among surgeons, it is well known that TT due to Graves diseases can be troublesome due to hypervascularization and inflammation of the gland, which makes it difficult to find the parathyroid glands and to preserve their blood supply.^(^ ^20^ ^)^

The observed overrepresentation of preoperative toxic thyroid diseases could explain some of the QoL impairment we report in chronic HypoPT patients. As expected, preoperative level of TSH was significantly lower in these patients, compared with nontoxic patients, which might affect the QoL itself.

### Concurrent hypothyroidism

Because chronic HypoPT in these patients is a result of unintended damage to the parathyroid glands during TT, these patients also suffer from a state of concurrent hypothyroidism. Hypothyroidism has been shown previously to affect QoL, despite being euthyroid on levothyroxine treatment.^(^ ^12^ ^)^ The influence of TSH on QoL in patients suffering from chronic HypoPT have previously been investigated,^11^, ^16^ ^)^ but the results still remain inconsistent.

In this study, all patients suffered from hypothyroidism and were receiving some kind of thyroid hormone supplement, most often being levothyroxine (97.6%).

We could not report an association between prospective TSH and the impact of QoL. Patients with dysregulated TSH were present in both groups (*p* = .94), and results did not change after adjusting for TSH (model 1). Because this study has no control group of healthy individuals we could not examine whether hypothyroidism itself has an impact on QoL. However, this study implies a combination of HypoPT and hypothyroidism to have a greater impact on QoL than HypoPT itself in accordance with comparable studies.^(^ ^11^, ^13^ ^)^ Furthermore, despite a higher comorbidity score among the patients without chronic HypoPT, their QoL scores were less affected. This indicated a strong importance of HypoPT disease on QoL in this study population.

### Biochemical fluctuations

Maintaining se‐ionized‐Ca^++^ within the therapeutic range remains a challenge in these patients.^(^ ^9^ ^)^ Previous authors have failed to find a correlation between biochemical markers such as calcium levels and QoL scores.^(^ ^16^, ^19^, ^21^ ^)^ As a result of the conventional treatment with calcium, vitamin D3 and active vitamin D, most of our chronic HypoPT patients presented with se‐ionized‐Ca^++^ levels within the normal range (mean 1.27 mmol/L).

Despite large amounts of supplements, we could confirm an insufficient effect on the physiological calcium homeostasis in the group of chronic HypoPT patients, because large variations and fluctuations were seen (range, 0.89–1.40 mmol/L). The one patient not receiving active vitamin D presented with the lowest ionized‐Ca^++^ (0.89 mmol/L). Our findings could not report a relation between calcium fluctuations and QoL outcome (Table 3).

### Lack of parathyroid hormone

Some authors have suggested the impairment of QoL to be a direct result of the lack of stimulation by parathyroid hormone,^(^ ^13^, ^17^, ^22^ ^)^ because receptors are expressed in numerous brain regions as well as in muscle tissue. This correlates with the impairment seen in the physical domains. The hypothesis has been studied in several clinical trials, investigating the efficacy of replacement therapy with recombinant human parathyroid hormone (rhPTH(1‐84)) on QoL. Some authors have reported an association between the number of treatment years with rhPTH(1‐84), a reduction of symptoms, and increasing QoL in the SF‐36 questionnaire.^(^ ^19^, ^23^, ^24^ ^)^

Other studies have not been able to prove any significant beneficial effects.^(^ ^18^ ^)^ There is, however, an agreement between researchers that rhPTH(1‐84) therapy seems to stabilize the se‐ionized‐Ca^++^ level and lower the need for supplemental calcium and vitamin D, when compared to conventional treatment.^(^ ^25^ ^)^

Whether the reported improvement in QoL is due to a more stable se‐ionized‐Ca^++^ level, a reduction in supplemental treatment, or a direct effect of parathyroid hormone still remains uncertain.

### Limitations and strengths

This clinical follow‐up study is one of few investigating QoL in patients suffering from chronic HypoPT after TT compared to well‐matched patients undergoing the to same operative procedure, at the same department.

The participation rate of invited chronic HypoPT patients was 54% and only included women, which contributed to homogeneous and comparable groups. Extending the recruitment period could have enlarged our sample size and thereby empowered the results further. The sample size might have been too small to illuminate some associations and might have overestimated others. Even though the RAND SF‐36 is validated for clinical use, it is an unspecific questionnaire that might result in HypoPT symptoms not to be reported in the domains.

## Conclusion

In conclusion, this study suggests impairment of QoL in more domains among patients suffering from postoperative chronic HypoPT after TT in the RAND SF‐36, compared with relevant well‐matched patients also undergoing TT. Neither a concurrent disease of hypothyroidism, other comorbidities, nor prospective biochemical values of TSH and se‐ionized‐Ca^++^ affected the QoL impairment. A preoperative diagnosis of toxic thyroid disease decreased the number of significant affected QoL domains. There is a need of better medical treatment and management of this group of patients.

## Disclosures

None declared.

## Author Contributions

**Camilla Jørgensen:** Conceptualization; data curation; formal analysis; investigation; methodology; project administration. **Preben Homøe:** Conceptualization; supervision; validation. **Morten Dahl:** Data curation; supervision; validation. **Mette Friberg:** Conceptualization; supervision; validation.

## Conflict of Interest

The authors have nothing to disclose.

### Peer Review

The peer review history for this article is available at https://publons.com/publon/10.1002/jbm4.10479.

## Supporting information


**Supplementary Table S1** Adjusted quality of life (QoL) scores by RAND‐SF‐36 questionnaire in patients with chronic hypoparathyroidism
