# Supplementary material for: Postoperative Chronic Hypoparathyroidism and Quality of Life After Total Thyroidectomy
Source: JBMR Plus. 2021 Mar 16;5(4):e10479. doi: 10.1002/jbm4.10479 (PMC8046100; doi:10.1002/jbm4.10479)
Supplement: Supplementary file 1 — Supplementary Table S1 Adjusted quality of life (QoL) scores by RAND‐SF‐36 questionnaire in patients with chronic hypoparathyroidism [file JBM4-5-e10479-s001.docx]

**Supplementary Table 1. Adjusted Quality of life (QoL) scores by RAND-SF-36 questionnaire in patients with chronic hypoparathyroidism**

| SF-36 variables | No Chronic HypoPT (n=28) | Chronic HypoPT + residual PTH (n=9) | Chronic HypoPT - residual PTH (n=5) |
| --- | --- | --- | --- |
| Physical Functioning | 82.0 ± 3.7 | 72.3 ± 6.2 | 57.6 ± 11.8 |
| Role Physical | 80.7 ± 6.1 | 44.2 ± 17.4 | 54.8 ± 14.6 |
| Bodily Pain | 77.2 ± 5.0 | **49.6 ± 9.8*** | 63.5 ± 17.3 |
| General Health | 46.8 ± 2.4 | **29.9 ± 4.4*** | **38.5 ± 3.1*** |
| Role Emotional | 85.6 ± 6.2 | 52.0 ± 14.4 | 78.2 ± 20.3 |
| Vitality | 58.1 ± 3.6 | **29.9 ± 10.1*** | **34.6 ± 10.6*** |
| Social Functioning | 85.9 ± 3.7 | 73.6 ± 9.0 | 66.4 ± 13.7 |
| Mental Health | 77.5 ± 2.8 | 69.9 ± 5.6 | **52.1 ± 5.6*** |

Values are mean ± sd. *P<0.05 vs controls. Differences in QoL score was assessed using ANVOCA models adjusted for age, ACCI, TSH, and ionized calcium.
